# Lactated Ringer Versus Albumin in Early Sepsis Therapy (RASP) study: preliminary data of a randomized controlled trial

**DOI:** 10.1186/cc14435

**Published:** 2015-03-16

**Authors:** C Park, E Osawa, J Almeida, R Nakamura, I Duayer, J Fukushima, G Queiroz, F Galas, L Hajjar

**Affiliations:** 1 ICESP, São Paulo, Brazil

## Introduction

Adequate fluid therapy is essential to the care of septic patients, aiming to optimize oxygen delivery without compromising microcirculation. In recent years, a few studies have suggested that albumin may be superior when compared with crystalloids in severe cases of septic shock. However, there are no data in the first hours of resuscitation. The aim of this study is to evaluate whether albumin 4% solution compared with lactated Ringer decreases 30-day mortality in cancer patients with septic shock.

## Methods

The Lactated Ringer Versus Albumin in Early Sepsis Therapy (RASP) study is a prospective, randomized, double-blind and controlled trial, with 360 patients. Until November 2014, at the Cancer Institute of University of São Paulo, we enrolled 110 patients with cancer and septic shock to receive as resuscitation fluid in the first 12 hours of ICU an admission bolus of albumin 4% solution or lactated Ringer. The primary outcome was 30-day mortality. Secondary outcomes include ICU mortality, ICU and hospital length of stay, 90-day mortality, daily SOFA score, rates and length of mechanical ventilation, renal replacement, needing of vasopressor drugs, status performance and fluid balance.

## Results

From 650 eligible patients, 110 patients were included in the study - 50 patients in the albumin group and 60 in the Ringer group. The mean age was 63 (57 to 70) years in the albumin group and 61 (51 to 71) in the Ringer group, *P *= 0.508. Most patients were male (58% in the albumin group vs. 56.1% in the Ringer group, *P *= 0.846). The ECOG was similar between the albumin and Ringer groups ((0) 26% vs. 8%, (1) 38% vs. 36.8%, (2) 20% vs. 38.6%, (3) 16% vs. 15.8%, *P *= 0.05). The SAPS 3 admission score was 51 ± 13 in the albumin group and 49 ± 10 in the Ringer group, *P *= 0.492. The total amount of administered fluid in the first 12 hours of resuscitation was 1,000 ml (1,000 to 1,500) in the albumin group and 1,000 ml (1,000 to 1,000) in the Ringer group, *P *= 0.59. The 12-hour fluid balance was 1,053 ml (385 to 1,700) in the albumin group and 990 ml (200 to 1,525) in the Ringer group. The 30day mortality was similar in both groups (60% in the albumin group and 50.9% in the Ringer group, *P *= 0.34). No significant differences in the other secondary outcomes were observed between the two groups.

## Conclusion

In cancer patients with septic shock, resuscitation with albumin 4% as compared with lactated Ringer did not improve the rate of survival at 30 days.
